# Supplementary material for: A convenient online desalination tube coupled with mass spectrometry for the direct detection of iodinated contrast media in untreated human spent hemodialysates
Source: PLoS One. 2022 Jun 6;17(6):e0268751. doi: 10.1371/journal.pone.0268751 (PMC9170114; doi:10.1371/journal.pone.0268751)
Supplement: S1 Fig — (A) and (B), Three endogenous metabolites along with the ioversol (m/z 807.87 and m/z 829.86) were detected in untreated spent hemodialysates of patients #2 and #3 at the beginning of CHDF. (DOCX) [file pone.0268751.s001.docx]

**
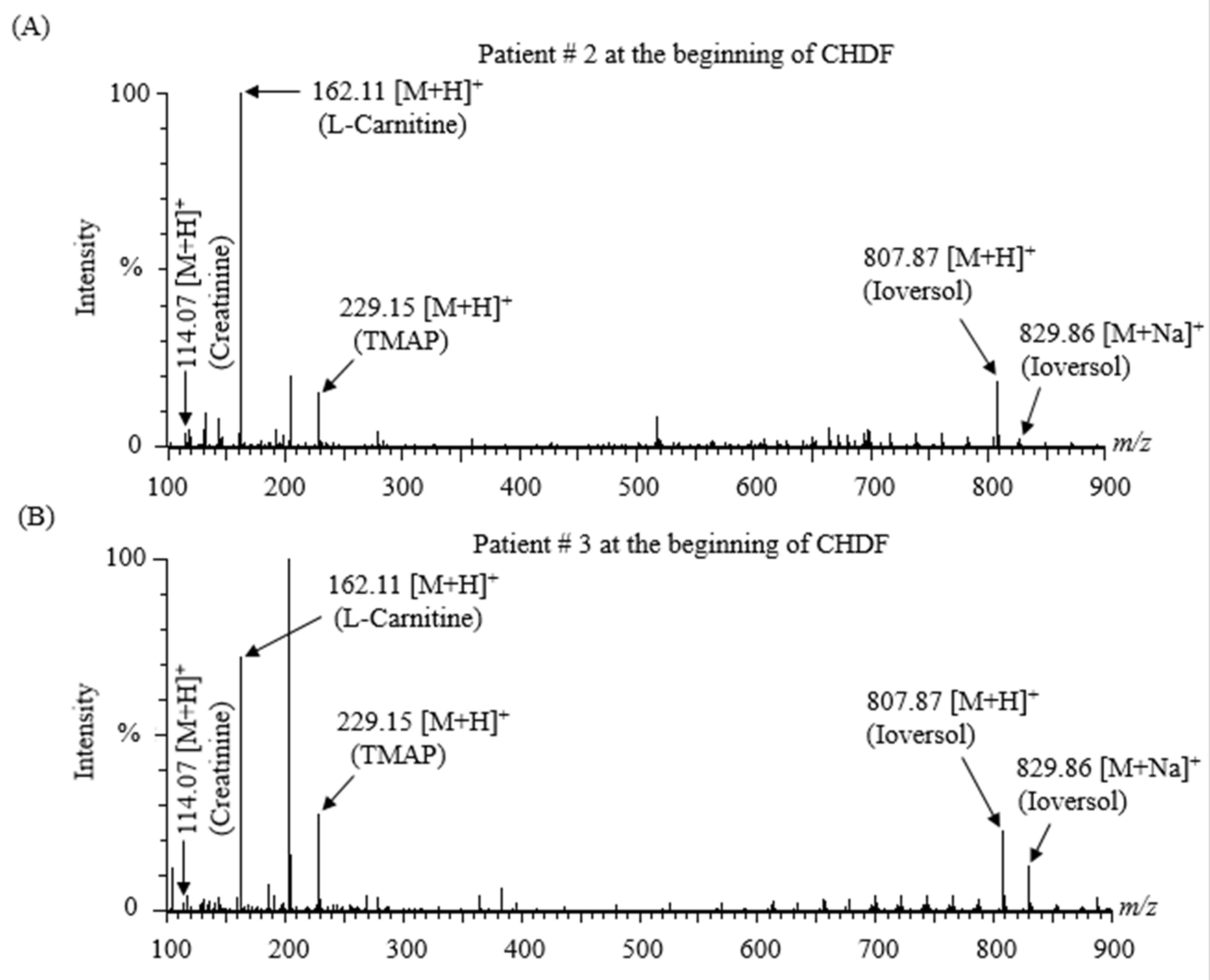
**

**S1 Fig. Detection of ICM and three endogenous metabolites in untreated spent hemodialysates of patients #2 and #3 by Synapt G2 Q-TOF MS.** (A) and (B), Three endogenous metabolites along with the ioversol (*m/z* 807.87 and *m/z* 829.86) were detected in untreated spent hemodialysates of patients #2 and #3 at the beginning of CHDF.
