# Supplementary figures and images for: A convenient online desalination tube coupled with mass spectrometry for the direct detection of iodinated contrast media in untreated human spent hemodialysates
Source: PLoS One. 2022 Jun 6;17(6):e0268751. doi: 10.1371/journal.pone.0268751 (PMC9170114; doi:10.1371/journal.pone.0268751)

**
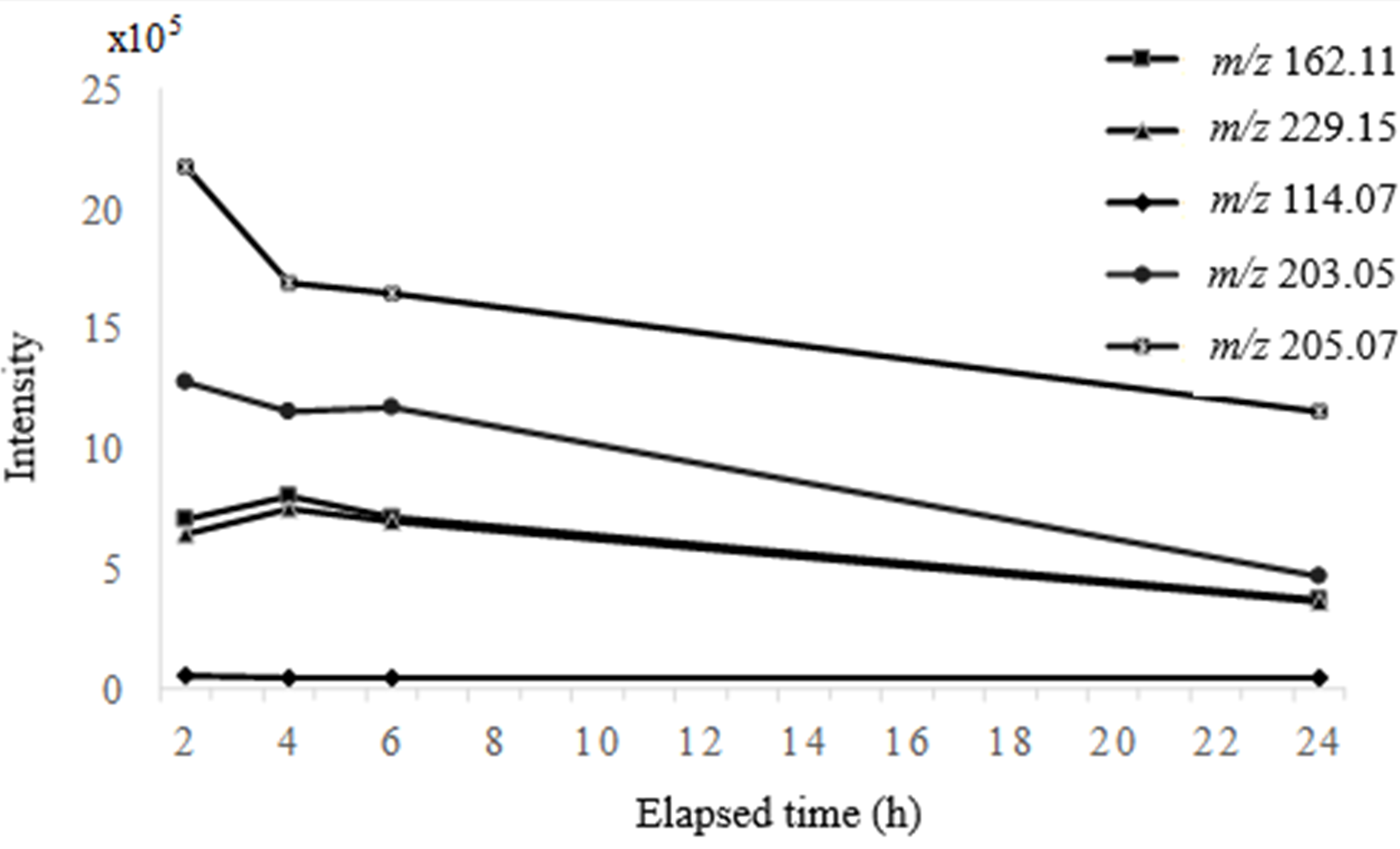
**

**S2 Fig. The changes of metabolites with time (2-24h) in spent hemodialysis of patient #1 during CHDF.**

Supplement: S2 Fig — (DOCX) [file pone.0268751.s002.docx]

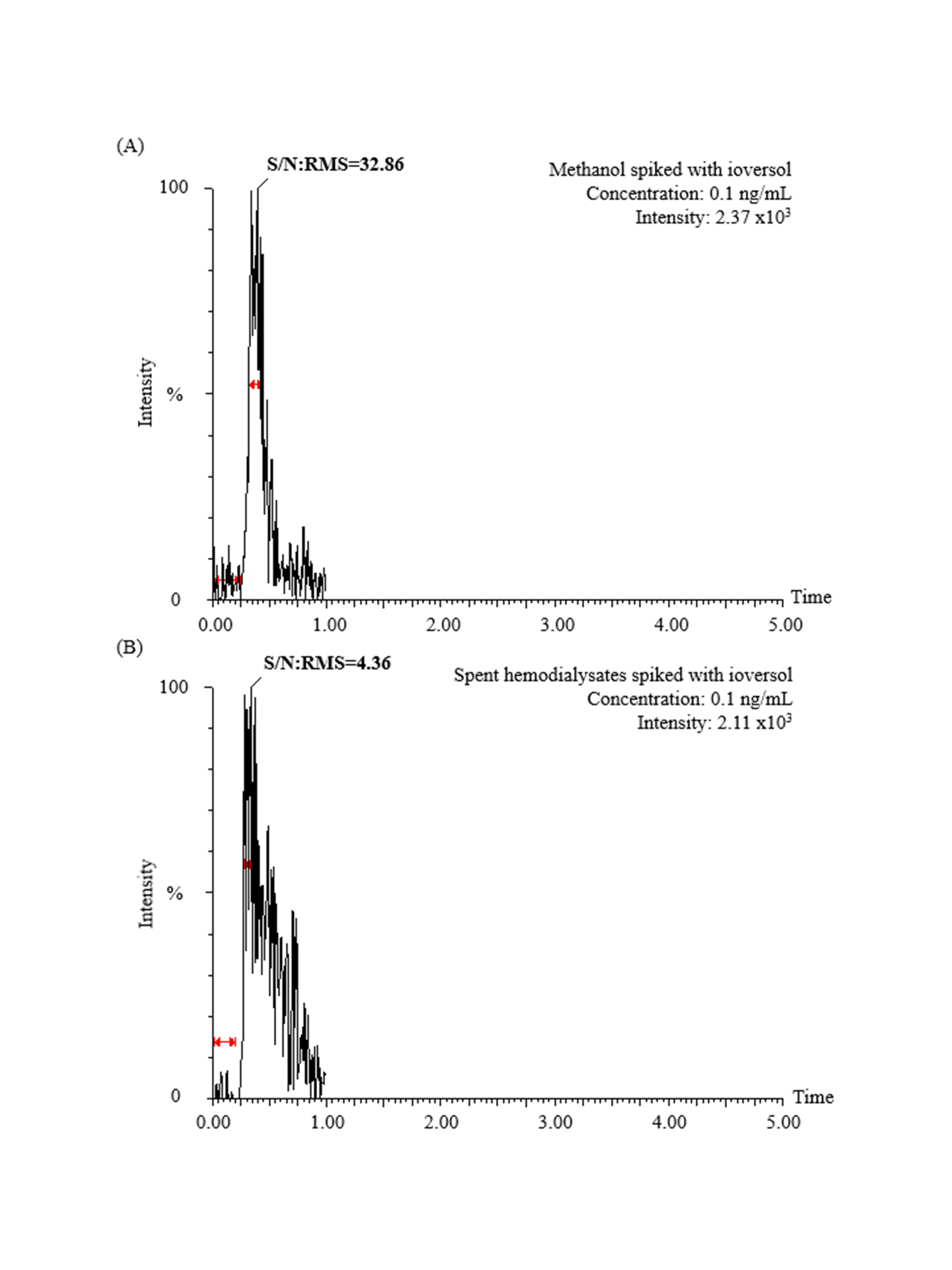


**S5 Fig.** The signal to noise ratio (S/N) of ioversol in methanol (A) and spent hemodialysates (B).

Supplement: S5 Fig — (DOCX) [file pone.0268751.s005.docx]
