# Supplementary material for: A convenient online desalination tube coupled with mass spectrometry for the direct detection of iodinated contrast media in untreated human spent hemodialysates
Source: PLoS One. 2022 Jun 6;17(6):e0268751. doi: 10.1371/journal.pone.0268751 (PMC9170114; doi:10.1371/journal.pone.0268751)
Supplement: S3 Fig — Patients #2 and #3 were found ioversol positive, denoted as (A) and (B), respectively. (DOCX) [file pone.0268751.s003.docx]

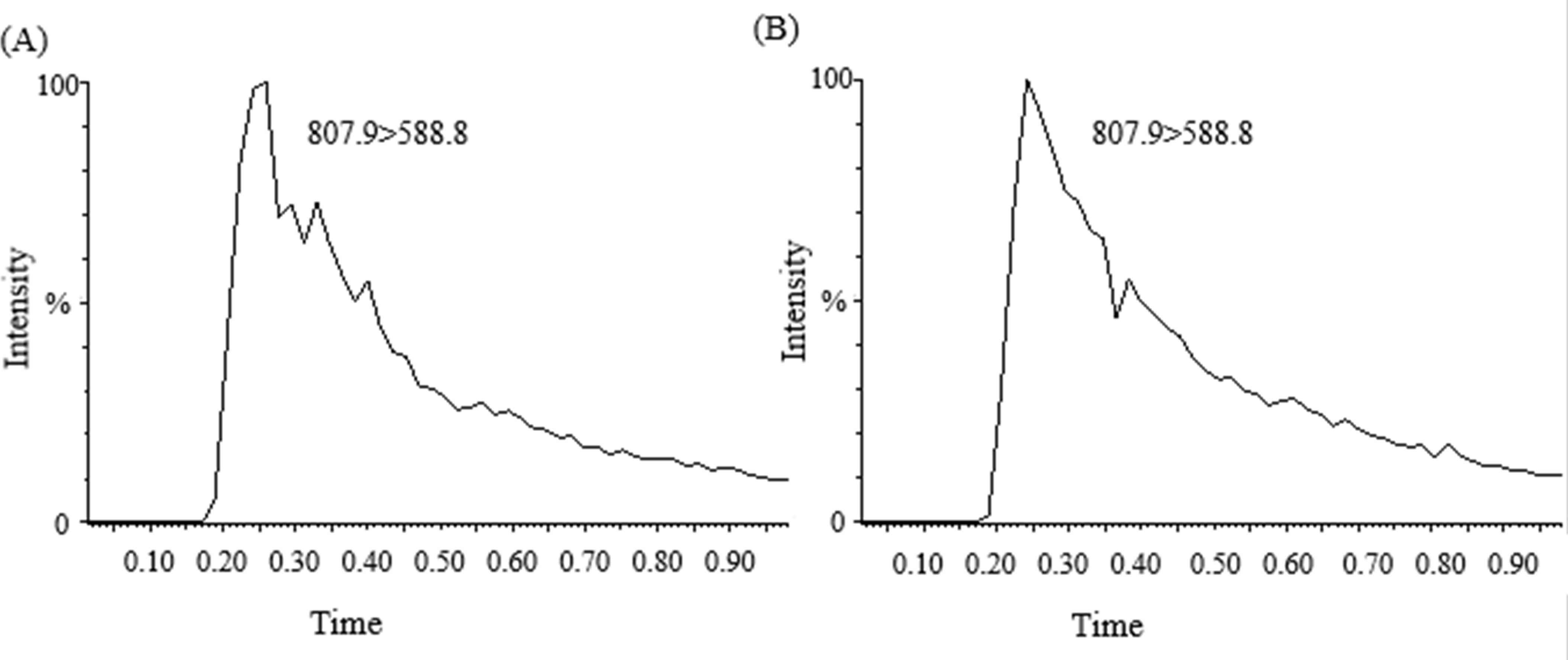


**S3 Fig. Detection of ioversol in untreated spent hemodialysates by MRM profiling.** Patients #2 and #3 were found ioversol positive, denoted as (A) and (B), respectively.
