# Supplementary material for: A convenient online desalination tube coupled with mass spectrometry for the direct detection of iodinated contrast media in untreated human spent hemodialysates
Source: PLoS One. 2022 Jun 6;17(6):e0268751. doi: 10.1371/journal.pone.0268751 (PMC9170114; doi:10.1371/journal.pone.0268751)
Supplement: S4 Fig — The left top denoted blank (methanol), whereas the right top denoted blank (spent hemodialysates), Later, we spiked with known amounts of ioversol ranging from 0.01 to 1000 ng/mL. Here, the lowest detectable concentration of ioversol is 0.1 ng/mL in spent hemodialysates or methanol. (DOCX) [file pone.0268751.s004.docx]

**
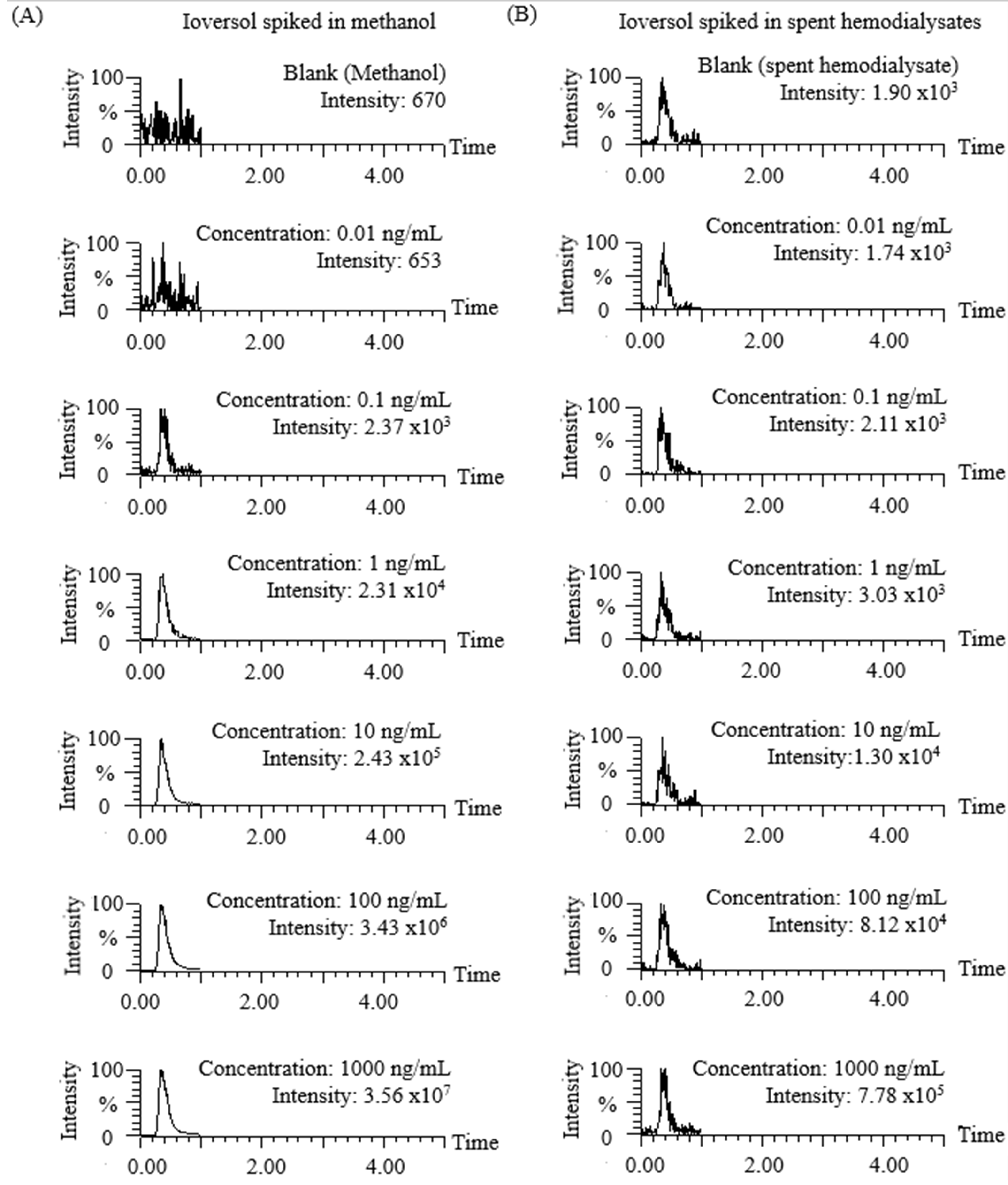
S4 Fig.** Determination of the limit of detection of ioversol in methanol (A) or spent hemodialysates (B). The left top denoted blank (methanol), whereas the right top denoted blank (spent hemodialysates), Later, we spiked with known amounts of ioversol ranging from 0.01 to 1000 ng/mL. Here, the lowest detectable concentration of ioversol is 0.1 ng/mL in spent hemodialysates or methanol.
