# Supplementary material for: A convenient online desalination tube coupled with mass spectrometry for the direct detection of iodinated contrast media in untreated human spent hemodialysates
Source: PLoS One. 2022 Jun 6;17(6):e0268751. doi: 10.1371/journal.pone.0268751 (PMC9170114; doi:10.1371/journal.pone.0268751)
Supplement: S6 Fig — (DOCX) [file pone.0268751.s006.docx]

**
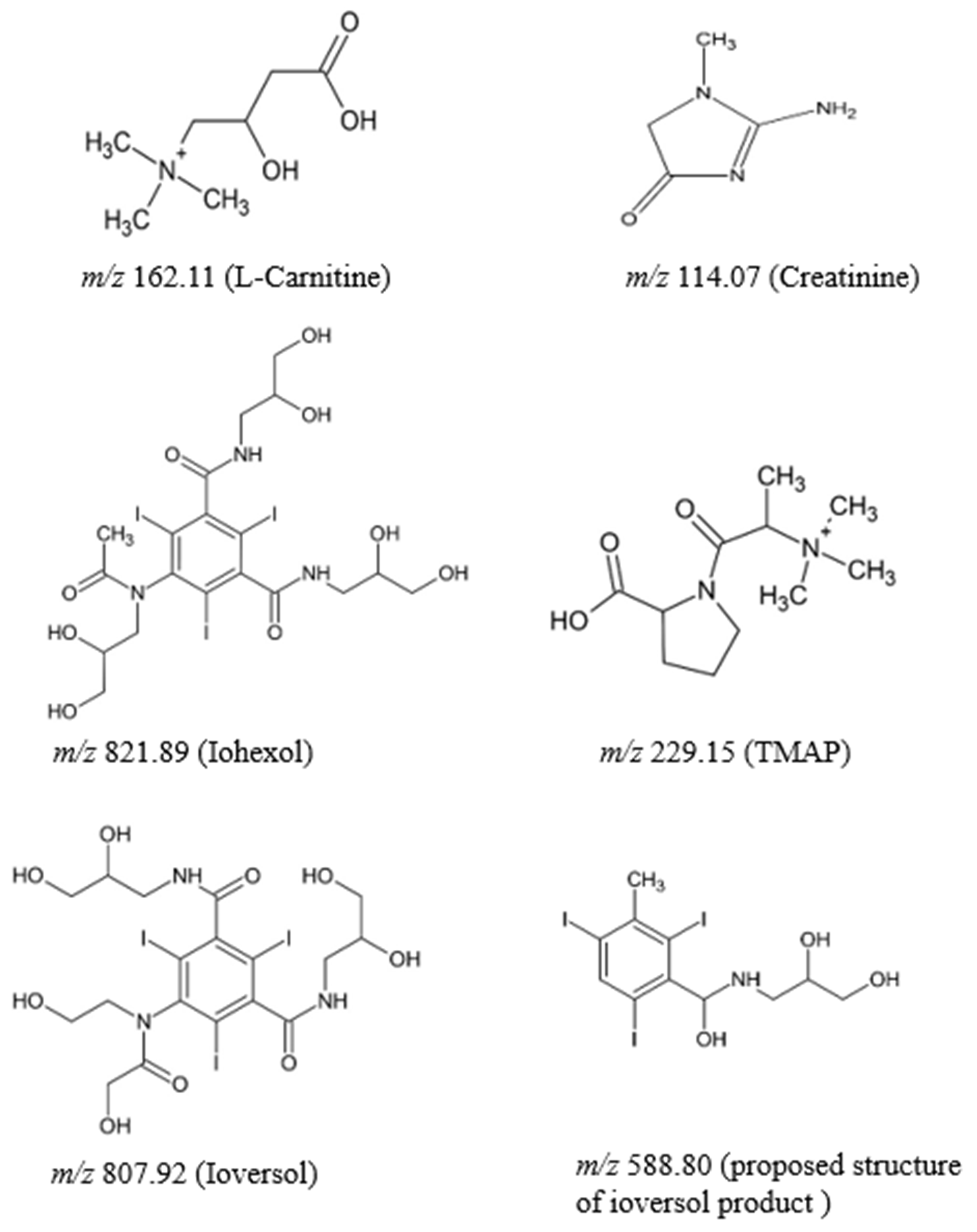
**

**S6 Fig. Chemical structure of the metabolites, ICM (iohexol, ioversol), and ioversol product detected in spent hemodialysates.**
