# Supplementary material for: A convenient online desalination tube coupled with mass spectrometry for the direct detection of iodinated contrast media in untreated human spent hemodialysates
Source: PLoS One. 2022 Jun 6;17(6):e0268751. doi: 10.1371/journal.pone.0268751 (PMC9170114; doi:10.1371/journal.pone.0268751)
Supplement: S1 Table — (DOCX) [file pone.0268751.s007.docx]

**S1 Table. Patient characteristics.**

| Patient characteristics | Patient #1 | Patient #2 | Patient #3 |
| --- | --- | --- | --- |
| Age (year) | 50 | 83 | 69 |
| Sex | Male | Female | Female |
| Height (cm) | 174 | 150 | 148 |
| Body weight (kg) | 89.8 | 50.6 | 83.0 |
| eGRF value (ml/min/1.73 m^2^) | 14 | 29 | 26 |
| Date of CT/ CAG | CT scan was performed during CHDF | CAG was performed one day before the initiation of CHDF | CAG was performed one and two days before the initiation of CHDF, respectively. |
| Contrast agent | Omnipaque 300 (Iohexol 64.7g/100ml) | Optiray 350  (Ioversol 74.1 mg/100ml) | Optiray 350  (Ioversol 74.1 mg/100ml) |
| Iodine content | 300 mg I/ml | 350 mg I/ml | 350 mg I/ml |
| Amount received | 150 ml | 30 ml | 70 ml and 100 ml |
| Route of administration | Intravenous | Intravenous | Intravenous |
| Diagnosis | Acute kidney injury, Crohn's disease, | Acute kidney injury, Ventricular septal perforation, Acute myocardial infarction, Stenosis, Stenosis of large arteries, Infarction of the brain | Acute kidney injury, Type 2 diabetes, Hypertension,  Lipid disorders, Hyperaemic optic disorder, Osteoporosis, Hyperuricemia, Spinal compression fracture, Uterine prolapse, Cerebral infarction. |
