# Supplementary material for: A convenient online desalination tube coupled with mass spectrometry for the direct detection of iodinated contrast media in untreated human spent hemodialysates
Source: PLoS One. 2022 Jun 6;17(6):e0268751. doi: 10.1371/journal.pone.0268751 (PMC9170114; doi:10.1371/journal.pone.0268751)
Supplement: S2 Table — (DOCX) [file pone.0268751.s008.docx]

**S2 Table. Detailed MRM transition and compound-dependent parameters.**

| SL. No | Compound | Polarity | Precursor  ion (*m/z*) | MRM transition  (*m/z*) | CE (eV) | Dwell time  millisecond (ms) |
| --- | --- | --- | --- | --- | --- | --- |
| 1 | Ioversol | + | 807.9 | 588.8 | 25 | 1.0 |
