# Supplementary material for: A convenient online desalination tube coupled with mass spectrometry for the direct detection of iodinated contrast media in untreated human spent hemodialysates
Source: PLoS One. 2022 Jun 6;17(6):e0268751. doi: 10.1371/journal.pone.0268751 (PMC9170114; doi:10.1371/journal.pone.0268751)
Supplement: S3 Table — (DOCX) [file pone.0268751.s009.docx]

**S3 Table. Comparison of the signal intensity and spectral signal-to-noise ratio (S/N) of ioversol with and without an online desalination tube.**

| Precursor ion (*m/z*) | Product ion (*m/z*) | Intensity  using desalination tube | Intensity  without desalination tube | Fold of intensity  difference | S/N  using desalination tube | S/N without desalination tube |
| --- | --- | --- | --- | --- | --- | --- |
| 807.9 | 588.8 | 2.45 x 10^8^ | 1.38 x 10^6^ | 177.54 | 1433.38 | 189.22 |
