# Supplementary material for: A convenient online desalination tube coupled with mass spectrometry for the direct detection of iodinated contrast media in untreated human spent hemodialysates
Source: PLoS One. 2022 Jun 6;17(6):e0268751. doi: 10.1371/journal.pone.0268751 (PMC9170114; doi:10.1371/journal.pone.0268751)
Supplement: S4 Table — (Numerical intensity value of Fig 5). (DOCX) [file pone.0268751.s010.docx]

**S4 Table. The signal intensity of ioversol (*m/z* 807.9) with time (0-24h) in spent hemodialysates of patients #2 and #3.** (Numerical intensity value of Fig 5).

| Elapsed time (h) | The signal intensity of  *m/z* 807.9 observed in patient #2 | The signal intensity of  *m/z* 807.9 observed in patient #3 |
| --- | --- | --- |
| 0 | 50192984 | 115805976 |
| 0.5 | 47908308 | 110944176 |
| 1 | 51360368 | 110419624 |
| 2 | 50829848 | 104281352 |
| 4 | 45052068 | 97523936 |
| 6 | 40170416 | 92170920 |
| 24 | 12410090 | 51738108 |
