# Supplementary material for: A convenient online desalination tube coupled with mass spectrometry for the direct detection of iodinated contrast media in untreated human spent hemodialysates
Source: PLoS One. 2022 Jun 6;17(6):e0268751. doi: 10.1371/journal.pone.0268751 (PMC9170114; doi:10.1371/journal.pone.0268751)
Supplement: S5 Table — (Numerical intensity value of S2 Fig). (DOCX) [file pone.0268751.s011.docx]

**S5 Table. The signal intensity of metabolites (*m/z* 162.11, *m/z* 229.15, *m/z* 114.07, *m/z* 203.05, and *m/z* 205.07) with time (2-24h) in spent hemodialysates of patient #1.** (Numerical intensity value of S2 Fig)

|  | Intensity | | | | |
| --- | --- | --- | --- | --- | --- |
| Elapsed time (h) | *m/z* 162.11 | *m/z* 229.15 | *m/z* 114.07 | *m/z* 203.05 | *m/z* 205.07 |
| 2 | 702979 | 648585 | 53599 | 1277586 | 2177334 |
| 4 | 801756 | 748209 | 45883 | 1152027 | 1694831 |
| 6 | 712990 | 694584 | 48422 | 1176402 | 1648595 |
| 24 | 374549 | 363182 | 48343 | 469890 | 1152814 |
